# Plural violence(s) and migrants’ transnational engagement with democratic politics: the case of Colombians in Europe

**DOI:** 10.1186/s40878-022-00298-w

**Published:** 2022-06-15

**Authors:** Anastasia Bermudez

**Affiliations:** 1 Department of Social Anthropology, Universidad de Sevilla, C/ Doña María de Padilla, 41004 Seville, Spain; 2 Centre for Ethnic and Migration Studies (CEDEM), Université de Liège, Liège, Belgium; 3 Instituto Universitario de Estudios Sobre América Latina (IEAL), Universidad de Sevilla, Seville, Spain; 4 GEISA (Grupo Para El Estudio de Las Identidades Socioculturales en Andalucía - GEISA SEJ-149), Seville, Spain

**Keywords:** Plural violence(s), Colombia, Democratic politics, Transnationalism, Migrants, Europe

## Abstract

This article explores how multiple, interrelated violence(s) shape the ways in which migrants relate to democratic politics transnationally. It takes as a departing point the literature on violent democracies and violent pluralism in the Latin American context, and more specifically the situation in Colombia, where democratic institutions coexist with plural violence(s). Following on from studies of migrant transnational politics, the analysis focuses on the Colombian diaspora and how migrants coming from violent democracies engage politically with the home country. Based on extensive research with Colombian migrants in Europe since the mid-90s, the article shows how despite different motivations for migrating, origin-country violence plays a significant role in the lives of many Colombians abroad. It then explores how violence influences migrants’ transnational politics. Migrating from a context of pervasive violence(s) can affect migrants’ sense of transnational belonging as well as increase mistrust and indifference towards formal democratic processes. However, the situation in the home country, together with being exposed to different conditions in the host society, can also motivate migrants to participate transnationally in initiatives to end the violence, thus increasing cooperation and trust.

## Introduction

Colombia is a complex country. Some commentators emphasize the strength of its political system based on liberal and formal democratic traditions, including an intense electoral record (Posada Carbó, 1999). On the other hand, critics associate this democracy with endemic clientelism, corruption and violence, as well as high levels of exclusion (Castillo-Ospina, 2013; Ramírez, 2010; Roldán, 2010). Thus, the country is celebrated as an island of economic and political stability within South America, while at the same time contributing some of the world’s worst records on violence and one of the longest internal armed conflicts in the region. It is also important to mention the role played by social violence, whether connected to the conflict, drugs trafficking, socioeconomic inequalities or the domestic sphere. Despite the signing in 2016 of a peace agreement with the largest guerrilla group in the country, violence continues to be part of people’s lives in Colombia with the emergence of new violent actors such as those included in the GAOs (*Grupos Armados Organizados*—Organised Armed Groups) and levels of asylum seekers fleeing abroad rising (Bermudez, 2021).

These different violence(s) are deeply interrelated and have had as one of their main consequences the displacement of millions of people, profoundly affecting the social fabric of the country (see, for instance, Celestina, 2016). Attention has focused foremost on the plight of the internally displaced population, amounting to more than 8 million people, the largest in the world according to official data (United Nations High Commissioner for Refugees [UNHCR], 2021), while the situation of refugees and others migrating abroad is less well known (see, for instance, Riaño-Alcalá and Villa 2008). This is partly because Colombian international outflows have been treated largely as economic migration, while the role played by different types of violence in this exodus has being obscured.

Still, Colombian migrants abroad are not merely the consequence of economic, social or political conditions expelling them from the home country; they are also active agents who have (re)created new lives in different host countries, forming transnational connections with the home country and within the diaspora, including in the political sphere. At the same time, the violence experienced, as Celestina (2016) argues, does not only have effects at the macro level, but also within communities and in people’s individual existence. The rest of this article explores the impact of different, interrelated violence(s) in Colombian migrants’ lives and how this affects how they relate to democratic politics in Colombia (and the receiving country). The analysis is informed by theoretical debates around the concepts of violent democracies and violent pluralism (Pérez-Armendáriz, 2021; Von Holdt, 2014), as well as earlier literature on political transnationalism and Colombian migration abroad, and based on extensive qualitative research with Colombian migrants in Europe (mostly United Kingdom [UK], Spain and Belgium) carried out since the mid-1990s until now (Bermudez, 2016, 2021), as detailed in the next two sections. After this, the text focuses first on how despite different motivations for migrating abroad various inter-related types of violence have played a significant role in many Colombian migrants’ lives. Secondly, it analyses the impact on experiences in countries of settlement, and more particularly on political perceptions and participation from a transnational perspective. Results show how violent democracies can have different effects, increasing mistrust and indifference towards democratic processes and within communities, but also encouraging participation in informal politics favouring peace. The article ends with the conclusions, linking findings with the theoretical framework.

### Democracy, violence and migration in Colombia

Von Holdt (2014) argues that rather than treating violence and democracy as antithetical to each other, in many countries of the Global South they have become mutually constitutive. In democracies experiencing high levels of conflict, violence can act as an alternative source of power through which to “preserve” or “contest” the ruling order (Von Holdt, 2014: 129). This is why it would be wrong to define these democracies as failed or incomplete (see also Arias & Goldstein, 2010). Such theories have been applied in Latin America, where some countries with long democratic traditions or on the way to democratization experience high levels of plural violence(s). For Arias and Goldstein (2010: 5) violence is “critical to the foundation of Latin American democracies, the maintenance of democratic states, and the political behaviour of democratic citizens”. Although these arguments have been criticized for providing a “disturbing” and “unethical” vision of democracy (Sluka, 2011), the tentative concepts of “violent democracy” and “violent pluralism” offer an interesting prism through which to examine Colombia.

As Ramírez (2010) and Roldán (2010) show, violence has played a key role in Colombian politics, economics and society throughout most of its democratic history. Violent pluralism emphasizes the idea of using violence to support or contest the status quo by a plurality of actors, from the more institutionalized (parties, officials, state authorities) at different levels (national, regional, local) to civil society (economic interests, guerrillas, criminal organizations) and individuals (Pérez-Armendáriz, 2021). This reflects the situation in Colombia in the last few decades, where state forces, left-wing guerrillas and right-wing paramilitaries have fought a war fuelled by drugs-related and other criminal and social violence(s).

Compared to other countries in the region, Colombia has not gone through prolonged dictatorship. Still, throughout the nineteenth and twentieth centuries, inter-party violence coexisted with the functioning of a formal democracy and regular elections. *La Violencia* (The Violence, 1946–1958) left hundreds of thousands of people dead and displaced. Caballero (n.d.) claims that this conflict was “a sum of many, diverse violences in small letters: political, social, economic and religious”,^1^ thus emphasizing the relationship between the different types. *La Violencia* led to a brief military regime followed by the institutionalization of political exclusion through the *Frente Nacional* (National Front). Even though this new, limited democratic regime officially ended in 1974, and the 1991 Constitution was celebrated as a major step towards a more inclusionary democracy, many expectations remained unfulfilled. This is especially so in the case of the multiple violence(s) affecting the country. Colombia has had the longest internal armed conflict in Latin America—a confrontation between the State, left-wing rural and urban guerrillas and right-wing paramilitaries with different histories and impacts across the country and population groups-, mainly rooted in the struggle for land and other economic resources as well as political power. Alfredo Molano, long-term Colombian analyst, argues that: “the formula of all forms of struggle… is general for everyone doing politics (…)They all do it” (Narváez, 2019: n.p.).

This portrayal can be extended to other types of violence. Through the 1980s–1990s, Colombia assumed notoriety as one of the most violent countries in the world, with Medellin as the symbolic global capital of crime. The violence and power of the drug cartels had political repercussions, contributing to the armed conflict, extending corruption and seeking to build their own political programme. It also had widespread social impacts, increasing fear and mistrust within society and multiplying different violence(s) among vulnerable sectors such as the young, urban poor (McIlwaine & Moser, 2001). Studies correlate this generalised violence with levels of urbanisation and poverty, the proliferation of arms and the lack of a state monopoly on the use of violence (Briceño-León, 2008). Waldmann (2007) uses the more controversial concept “culture of violence” to explain the widespread use of violence by multiple collective and individual actors to resolve conflict, associated to high levels of impunity. For this author, although economic and political factors are important, they are not sufficient explanations for the levels of violence in the country. He sees violence and crime as a permanent (chronic) characteristic of Colombian society, and relates this to culture in its broader and narrower meanings. For him, violence and coercion have become “fixed components of [the] social and political machinery” and are perpetuated by collective values and norms (Waldmann, 2007: 594). This is reflected in the excessive use of violence, the entrenchment of a “friend-foe mentality” and the “the lack of restrictive taboos and informal sanctions against the unauthorized use of violence” (Waldmann, 2007: 598).

As a result authors speak of Colombia’s democracy as “deviant” (Murillo Castaño & Osorio Ramírez, 2007), with formal processes such as elections coexisting with plural violence(s) and a strong state focus on maintaining order (Ramírez, 2010). Initiatives ranking countries on the quality of their democracy, political rights and civil liberties or the strength of the state find Colombia lacking in important aspects, including it among “flawed democracies” (Democracy Index)^2^ or “partly free” countries (Freedom in the World).^3^ Furthermore, there is evidence of violence being used by all actors in Colombia (including the state) to influence the results of elections or eliminate opposition (Roldán, 2010).

Parallel to this, Colombia has gone through several peace processes with a multiplicity of armed actors, culminating in the peace accords with the Revolutionary Armed Forces of Colombia (FARC—*Fuerzas Armadas Revolucionarias de Colombia*) in 2016. This brought the country closer to a peaceful solution to the armed conflict (Moreira et al., 2015), however, reports are that violence is increasing again, particularly affecting community and environmental leaders as well as social activists. This context has left a legacy of deaths and personal injury, disappearances, human rights abuses, forced displacement and spoliation. At a more symbolic level, it has destroyed communities and undermined values of trust and solidarity, contributing to the degradation of political and social life, with different consequences across regions and sectors of the population. Celestina (2016: 105) talks about the impacts of exposure to violence on both the “politics of belonging” (related to citizenship) and “place-belongingness” (feelings of home). On the other hand, “a variety of democratic institutions (civic action committees, cooperatives, municipal action groups, peasant producer associations, etc.) have been promoted by sectors of the Colombian state and civil society as an antidote to violence” (Roldán, 2010: 67). Examples of this are the *zonas de reserva campesina* in rural areas, despite the difficulties faced by some of these communities in maintaining autonomous development and peace (Quijano-Mejía & Linares-García, 2017), as well as the wider role of civil society initiatives in the construction of peace (Ardila Muñoz, 2004).

Violence has affected worse the most vulnerable, including women and children, rural communities, indigenous and black populations, and the urban poor. Gill (2014: 54) exposes the damage and transformations suffered by working-class neighbourhoods in Barrancabermeja affected by paramilitary and criminal violence: “[s]ocial practices that once formed the integument of social life” disappeared or were badly affected, and people did not know who to trust any more, thus turning “inward” and seeking “individual solutions to their problems”, as mistrust, uncertainty and clientelism based on fear and the need to survive enforced new social and political norms. Other studies of poor urban communities in Colombia affected by economic and social violence have also found a significant erosion of social capital as a result of fear, disunity and a culture of “silence”, with distrust of state institutions particularly high (McIlwaine & Moser, 2001). Regional comparisons show Colombia to be among the top countries where citizens mistrust political parties, institutions and processes, related as well to perceptions of corruption (Carreras & Irepoglu, 2013), which could help explain the relative low levels of electoral participation.

However, we know less about the effects of plural violence(s) on Colombians abroad, and the impacts on their political attitudes and participation, both in formal and informal activities. Official data put the number of Colombian migrants living abroad in 2020 at just over 3 million (UN International Migrant Stock),^4^ while the UNHCR for this same year cites just under 190,000 Colombian refugees under its mandate and 70,917 asylum-seekers.^5^ The relative weight of the internally displaced population has meant that those crossing international borders have received less attention. Studies have looked at the many impacts of internal displacement, including processes of uprootedness and reconstruction, disruption of the social fabric and transformation of identities (Meertens, 2002), or the powerful presence of fear in the narratives of internally displaced people and refugees (Riaño-Alcalá, 2008). Some research also underlines the high levels of disunity, mistrust and exclusion within Colombian migrant communities abroad, in great part linked to the context of violence(s) in the home country (Guarnizo et al., 1999), which according to Guarnizo et al. (2003) contributes to a prevalence of private, individual transnational links over public, collective ones. While other work focuses on the different ways the Colombian diaspora abroad contributes politically to democracy in the home country (Bermudez et al., 2017).

The present article seeks to analyse in more depth the impact of multiple, interrelated violence(s) on Colombian migrants’ perceptions and actions towards democratic politics in the home country. It does so based on the wide literature on migrant transnational politics. Transnational politics generally refers to political activities that criss-cross national borders, and in this case to migrant political involvement in home countries as well as sending states´ policies influencing them (Martiniello & Lafleur, 2008). Practices can include a wide range of phenomena like “transnational election campaigns and cross-border voting, migrants’ rallies against injustices in the country of origin or demonstrations to defend it, or engagement in hometown associations” (Østergaard-Nielsen, 2003: 761). This relates as well to transnational citizenship, a highly contested concept that for the purposes of our analysis includes both conceptions from above (rights) and below (membership or belonging) (Fox, 2005; see also Celestina, 2016).

## Methodology

The analysis below is the result of more than 20 years researching Colombian migration to Europe, especially the UK, Spain and Belgium.^6^ These are among the main destinations for Colombian migrants and refugees in this region: the UK started to receive significant Colombian inflows from the 1970s, while Spain did not become a main destination for Latin American migrants until the 1990s and the start of the twenty-first century, but hosts by far the largest Colombian community in Europe; Belgium, by contrast, is home to a much smaller Colombian population, however, the research conducted unearthed significant transnational connections between these three communities, as explored in previous studies (Bermudez, 2016, 2021). Research involved mostly qualitative ethnography since the mid-1990s as part of different projects, amounting to more than 200 interviews with Colombian migrants and refugees, as well as informal conversations, interviews with key informants, participant observation, virtual ethnography and surveys.

The work here is based on qualitative data collected up until 2021. Although the different projects focused on diverse aspects of Colombian migration to Europe, such as the experiences of women and refugees or processes of integration in host societies, the issue of Colombian migrants’ transnational politics in the context of violence and search for peace in Colombia has always been a key part of the research, so it is possible to undertake a longitudinal analysis of the data. The objective is, first, demonstrate the connections between plural violence(s) and Colombian migration abroad, and, secondly, explore some impacts on migrants’ transnational politics in the context of Colombia’s violent democracy. To achieve this, the article builds up from previous analyses, incorporating more recent data and developments.

### Violent pluralism and migration from Colombia

Colombian migrants provide many, interrelated motivations for migrating, including economic, study or relationship motives. Violence is also a key direct or indirect factor, but not always acknowledged in research, with migration outside the region seen primarily as economic, in opposition to internal or cross-border displacements. Following a systematic analysis of all the qualitative interviews conducted, it was possible to detect three main ways in which experiences of plural violence(s) and their connections to mobilities come up in the narratives.

First, there are Colombians who left the home country for reasons directly related to the armed conflict and consider themselves refugees, whether they are recognised as such or not. Colombian refugees started arriving in Europe in the 1970s, first escaping state violence and later affected by different armed actors including paramilitaries and guerrillas. Two contrasting experiences within this group are those of Alfredo and Beatriz,^7^ with many others in between. Alfredo, a left-wing activist from Valle del Cauca who arrived in London in 1993 and was granted asylum, represents the classical political refugee:

I participated in a left-wing political party, was totally opposed and critical of the government. I organized [theatre] workshops in working class neighbourhoods… we were detained and I spent four years in prison (…) accused of being… a leader in a guerrilla group (…) I came out, and was detained again eight months later, when participating in a 1^st^ of May march… I had a lot, a lot of support from human rights organizations… Amnesty International recognized me as a political prisoner. So they gave me the ticket and I came.

A direct victim of state-led political violence, Alfredo had a week to prepare and leave with his family for London, a place they did not know. The experience was deeply traumatic and caused a strong rupture in his life. Interviewed in 2005, Alfredo had rebuilt his life, worked for a refugee organisation and continued his activism transnationally, seeking to improve social and political life in Colombia, but felt despondent and wanted to return. His case shows as well the difficulties inherent in transnational activism, as explored in the next section.

By contrast, Beatriz, who arrived from Cali in Madrid in 2001, followed later by her husband and children, left Colombia because of guerrilla violence and 4 years later (during the interview) was still waiting to regularize her migrant status. In Colombia, they owned a small business, and when they could not keep paying ‘taxes’ to the FARC guerrillas, they were threatened, prompting her displacement:

over there we had buses, we had a taxi… (…) At the time we had good money, and then they started applying pressure… the guerrillas… they started to ask us for money… until it becomes, well we didn’t have any more to pay… I told him [her husband] that I was coming to Spain… he did not want to leave... then I came, without knowing where I would end up, what I was going to do or anything.

When her husband joined her a few months later, after losing everything to the guerrillas and common criminals, they applied for asylum and 2 years later brought their children. But Beatriz was exposed to other violence(s) as well. Relations between the couple were already strained in Colombia, and there had been instances of domestic violence. When it restarted in Spain, she finally separated from him. This affected her negatively at different levels: while her husband obtained a residence permit through a regularization programme, she was still waiting; and she was the main person responsible for their three children, both financially and in terms of living arrangements, which made it harder to achieve economic security. Although she admitted to not having had much political involvement in Colombia, her precarious situation in Madrid did not leave any space or time for anything else. Her case shows how other types of violence like intra-familial abuse can become obscured by more public violence(s), but in fact are interrelated, with women often being more vulnerable. For Celestina (2006: 101), exposure to a decades-long conflict can change perceptions of “normalcy”, making (any type of) violence “an everyday cultural fact” (see also Waldmann, 2007).

Second, there are Colombian migrants affected by the conflict and other violence(s) indirectly or those who mentioned violence as a secondary factor for their migration. Their stories are very heterogeneous and include experiences like that of Tomás, a journalist who moved to London in 2001 with a student visa because he “needed a change”. Later during the interview, he explained that this was the result of the pressure he and his colleagues, working as war reporters in some of the main hotspots in Colombia, received from armed groups: “the threats and different pressure applied by armed groups, including guerrillas, paramilitaries and drug traffickers, and even the state […] our salaries were not worthy enough to risk our lives”. However, Raquel, in Brussels since 1990, left her neighbourhood in Medellin tired of the violence of the drug mafias and police repression, but also to further her studies and offer her daughters better options: “I had to live in a place that wasn’t like this [her previous neighbourhood]”. In other cases, people left Colombia escaping violence from unknown perpetrators, as it happened to Rocío, a school teacher from Cali who found herself in trouble after a colleague disappeared and was found dead. Rocío became the target of threats and persecution by unknown actors, leaving for the UK with her husband in 1997, where their application for asylum was denied because they could not prove who was after them or why:

she [her colleague] was transporting weapons… and in one of this instances, a packet went missing, and well, she was never seen again… I began to look for information, but when the threats got very close, well, they were following me and they wanted me to give them money… asking if I wanted the same thing that happened to my friend to happen to me… We looked for help… and were told that no one could do anything because in Colombia most people live under threat by the guerrillas, paramilitaries or common criminals… that the best option was to leave the country for a while.

What these stories have in common is how different types of violence by various armed actors contributed to diverse types of displacement, affecting ever wider sectors of Colombian society as violent pluralism expanded throughout the 90s and beginning of the new century; during this time of growing insecurity migration abroad became an increasing exit strategy (Guarnizo, 2006). All of these violence(s), as seen next, will have an impact on levels of mistrust within the migrant communities, and in terms of rejection of politics, although not affecting everyone equally.

Third, there are Colombian migrants who arrived in Europe to study, look for work opportunities, progress economically or join their loved ones. This group is the largest and most heterogeneous, coming from all parts of Colombia and social groups, and the majority of research has focused on them. Although violence is not a main part of their narratives, sometimes they talk about how it influenced their lives or that of their families in Colombia. Stephanie, who migrated to the UK in 2002 with her British husband explained that her main purpose for emigrating was to progress economically and help her family in Medellin, but being abroad has allowed her to see the situation in Colombia differently:

I lived in a neighbourhood where every day they killed lots of people there, and they would tell us that it was the war between gangs… we got used to it, to be, well, running all day when shots were heard, also to not be able to sleep because many times the bullets would pierce the walls… we never tried to do anything […] and paying a tax, every eight days they came, well they said it was to, to look after the neighbourhood.

Although people like Stephanie, from working class and poor urban areas, have been among the worst affected by plural violence(s), the wider impacts have touched every part of the country and sector of society. Thus Alba, a young woman from Bogota who moved to London with her husband in 2005 to complete their postgraduate education, decided to stay partly because her area of work, development and social issues, was a dangerous profession in Colombia, as she knew from previous experience: “we worked in projects that hit a nerve with the large economic groups, that are in fact the ones financing the political system. Then you start running risks”. Even interviewees not closely affected by the generalised situation of violence and insecurity, can find it emotionally draining, as Macarena, who arrived from Cali in Madrid in 2002 to study after obtaining a scholarship, exposes: “The social situation, the political situation in my country. It was very sad for me, it was the time when drug trafficking was causing atrocities… emotionally I felt sad”. Macarena makes a connection with her personal circumstances in Colombia at the time, which included a traumatic break up: “I ended a relationship, it was very hard… I wanted to leave the country, start again from zero”, somehow underlining the linkages between emotional turbulence in the private and public spheres.

As well as these cases, in many of the interviews and conversations with Colombian migrants and refugees, there are frequent references to other types of violence affecting their families and communities, including in the past, offering a glimpse of a continuum of violence framing their lives. Bernardo, a refugee in Brussels since 2002, tells the story of how as a child, during *La Violencia*, his family had to flee from their land in the Tolima region, suffering several rural and urban internal displacements until as a young man he moved to Bogota to study. Later, his work as a student leader, lawyer and member of a left-wing political party forced him to leave the country several times, until settling in Belgium. While Blanca, who joined her husband in Madrid from Medellin in 2001 to progress economically, explains how the violence arrived in her small town of origin, in the coffee-growing area, affecting everyone: “everyone wants to have power… the *sicarios* [hired assassins], paramilitaries, guerrillas, they just keep forming armed groups”.

These stories reflect the myriad ways in which plural violence(s) are crucial to understand displacement, not only internally but also abroad, as well as the day-to-day lives of these migrants and refugees, and their transnational political involvement. Levels and types of participation in democratic politics from abroad are going to depend, up to a point, on individual factors (as many other studies reflect) as well as migrants’ political capital, migration experiences and home and host contexts (Bermudez et al., 2014), but also, as explored next, on the impact of these intersecting violence(s) on trust and belonging.

### Trust, belonging and transnational politics

Exposure to plural violence(s) has had diverse impacts on the day-to-day lives of Colombian migrants in Europe, both in relation to the home and host countries. In his work in Barrancabermeja, Gill (2014: 32–33) mentions how violence led to new “silences, ruptures, understandings, and ways of living” and the fragmentation of previous solidarities. Studies among the internally displaced also reflect the “fear, mistrust, silences” and “avoidance” of the past caused by these experiences, as well as the impact of violence on solidarity networks and conflict resolution (Zuluaga, 2016: 72–73). Nevertheless, as mentioned earlier, even in these unfavourable circumstances, there are instances of collective resistance. Fear, mistrust and fragmentation can affect relations in the communities affected as well as their new places of settlement, but also more wider as people fleeing violence are received with suspicion. For Colombians displaced or migrating abroad, they might gain in terms of lesser exposition to violence, but at the same time have to confront a more unfamiliar (and sometimes hostile) environment. Research with Colombian communities abroad emphasises how these are crisscrossed by strong mistrust and divisions that prevent solidarity, both in their day-to-day workings and in transnational connections, affecting political participation (Guarnizo et al., 1999). This is blamed on class hierarchies, regional differentiations and plural violence(s) in Colombia, as well as the stereotypes faced abroad. However, these studies do not explore in depth how violence continues to affect daily lives and the impact on belonging and democratic politics.

Many migrants and refugees in Europe talked about feelings of (in)security that reflect the deep impressions left by violence, and how being abroad helped to ‘de-normalise’ it. As well as the case of Stephanie above, who was only able to see fully the violence she was exposed to in Medellin from the calmness of London, others like Javier, a journalist from the same city who fled to Madrid for the second time in 2001 due to threats to his life, recalls early experiences abroad that underline this:

friends invited me to a country house… we were on the road and I felt like a sense of anguish, and I didn’t know why… on the way back I realised something, that the anguish was due to the road we were in being empty, and here, it is ok, but in Colombia an empty road means that something is going on… you get used to living with those fears… and you live it as if it is a very normal thing.

Macarena, mentioned before, also talks about what the security of living in Spain, despite not earning enough to maintain herself and her son, means to her: “to go to… a park, the *Retiro*, with my son, feeling at ease because there is not going to be a killing, nothing is going to happen to him because of a lost bullet”. Thus, as Celestina (2016: 106) argues, security for people exposed to long-term, pervasive violence “involves a subjective dimension”. Being in contact with other co-nationals in the diaspora, especially if they do not know them well, can be seen as a potential threat to such security, while in other cases it can offer comfort and support, depending on histories of violence and conditions in the host country. For Roy, in Brussels since 1999 after joining his wife, who emigrated looking for work, and with a secure migrant status, relations with other Colombians are important, they help him feel closer to his culture and mitigate the “sadness that there is in this country”: “At the beginning there was a lot, a lot, a lot of coming together… to organise a party to help one person or another who was in a bad situation (…) I like being… with my own people, my people, with Colombians”.

It seems that in contexts where cultural differences with the home country are perceived as greater, and the migrant community is smaller, like in Brussels, they tend to rely more on each other. Colombians in London also speak of a greater sense of community at the beginning, when they were a smaller group consisting partly of refugees collaborating with other Latin American exiles: “now we are not so united, we are disunited, there are lots of people, lots of problems… and people don’t want to mix with each other” (leader of Latin American Organisation, 2006). As migration increases, more divisions appear, between refugees and economic migrants, between different types of refugees fleeing diverse violence(s) or along class and other social markers, and as the situation in the home country deteriorates, mistrust, fear and silences travel within the diaspora. Margarita, who arrived in Spain in 2005 after living in another country first, left Colombia (Bogota) originally to travel and earn money. In her first destination abroad the Colombian community was small and people felt close to each other, especially the women, most of whom worked in domestic service. In Spain she keeps in contact with friends from before spread over different cities now, but is careful not to get together with other Colombians or participate in community activities for two main reasons: there are “very dangerous people” and others involved with drugs within the community, and because she does not have a regular migrant status and fears deportation. The sense of mistrust and fear can be more potent among those fleeing violence, given suspicions that other Colombians can be part of the armed group behind their displacement. A fear that Isaac, in Madrid, half-jokingly calls “paranoid” but that has been backed by cases of people receiving threats or being monitored in exile (Bermudez, 2021). Isaac, from Medellin, left Colombia in 2001 because of his left-wing political and artistic activities, after becoming a target of paramilitary threats. In his narrative, he talks about how *La Violencia* impacted on his father’s family, originally from a rural area, and how levels of criminal violence make it even harder to live in his country, thus interlinking different types of violence: “you don’t know who is going after you, it could be your own neighbour, a relative”. This situation translates into general mistrust abroad:

I don’t have many relations with [Colombians here]… sadly we have a very bad reputation… there is another thing, I have been told to be very careful, being told to try and not go to the embassy or the consulate… and when I am asked by someone from Colombia, try not to tell them much about my history, because maybe the tentacles of those people reach here.

The multiple violence(s) experienced leave Colombians abroad in an ambiguous belonging situation, since for most their real home is back ‘there’, even when they have lived abroad for a significant period and acquired other nationalities (Bermudez, 2020), but at the same time return is not considered an actual (rather than wished) possibility (Bermudez and Paraschivescu, 2021) even when facing severe socioeconomic insecurity in the host society (Bermudez, 2019). These ambiguities have a reflection on transnational politics (Bermudez and Paraschivescu, 2021). Arias and Goldstein (2010) make a connection between violent pluralism and the quality of citizenship, presenting how extreme levels of different violence(s) in many Latin American formal democracies result in unequal access to citizenship and exclusion, which can be understood here in reference to both “state-based” and “society-based” notions of citizenship (Fox, 2005). More particularly, they focus on how the politics of violence affects “lived political experience” and its impact on “political practice and subjectivity” (Arias & Goldstein, 2010: 4). Taken into account both formal and informal political practices, we can explore this in the case of the Colombian communities in Europe.

First, if electoral politics is to be taken as an indicator of the health of a democracy or a reflection of citizen behaviour, then Colombia does not fare well. Despite its claim to have one of the most stable electoral records in the region, abstention rates are comparatively high(voting is not compulsory), especially in the case of the ‘external vote’ (Escobar & Gómez Kopp, 2015). Although there are many factors explaining electoral abstention from abroad, including administrative and bureaucratic barriers and migrants’ living conditions, among many interviewees in Europe mistrust and rejection of politics was important, especially among those with lower political capital and histories of violence and exclusion. There are cases of both continued silences and ruptures, with some Colombians feeling outside formal politics or overtly rejecting elections as reflections of the corruption, exclusion and violence experienced (“nothing changes”), while others found themselves disconnected from the political realities of the country while abroad, purposely or due to their circumstances:

in Colombia, to be a good politician, one has to be, let’s say corrupt… a friend of mine entered politics… since he didn’t let himself be corrupted, he was killed (Vicente, from Trinidad, a small town in Valle del Cauca, in London since 2014 after living in Spain first, looking for better economic opportunities);

No, never, ever, ever [voted in elections] (…) when there is such a huge level of corruption, it doesn’t matter… so I have never done it (Dylan, from Medellin, in Brussels since 2012, having arrived from Spain escaping the economic crisis, where he moved to travel and work);

In Colombia I participated in lots of marches, which had to do with drug trafficking issues (…) corruption, *Uribismo* [support for former president Uribe], paramilitaries, etc. However, I have never voted (Rufo, from Bogota, in Madrid since 2010, where he arrived to join his Italian wife).

Both Vicente and Dylan represent cases of migrants who left the country in search of opportunities they could not find in Colombia, while the former makes a direct link between politics and violence. Also, for Dylan and Rufo there is continuity in their disconnection from electoral politics in Colombia, but not from politics in general, since they confirm their interest in what happens in the country and have experience of participating in informal politics (marches, etc.). Moreover, Rufo explains that his views about formal politics, after his experiences in Spain during the 2008 economic crisis, are changing (he is becoming more involved), highlighting the importance of contexts of settlement:

right now I am following *Podemos* [new left-wing Spanish political party] (…) little by little I feel like a political being, like all the people who start waking up to a conscience that, let’s say, I didn’t have before… in Colombia, in general, there is like a huge discrediting of politics … democracy in Colombia doesn’t work… because of that, I was never interested in participating, beyond demonstrating against things I did not agree with, but… I never got interested in voting.

Continuity is also common among interviewees who voted and participated in electoral politics in Colombia and abroad, since they see it as a right and/or duty. For others, by contrast, being away from the home country encouraged disconnection. This rupture can be the result of a desire to leave behind past violence(s), as for Manuel and Pepa, a couple with two children from the coffee-growing area (*eje cafetero*) who sought asylum in Madrid (she arrived in 2000, and he and the children the following year), after their lives were in danger following Manuel’s election as town councillor. Interviewed 5 years later, through their narrative there is ample evidence of the sense of security gained, and the fear of losing this. Pepa explains that for them “security is fundamental”, that at least their lives are safe. Manuel adds that they are still experiencing the “secondary effects” of what happened to them in Colombia and as a result have abandoned all political work: “I have separated myself [from all that] (…) we are very isolated… very resistant to participate in things”.

At the same time, for other Colombians in the diaspora, contributing to solving the problems in the home country, including violence, is important. A strong sense of home and citizenship belonging (Celestina, 2016) can spur transnational political participation in formal and informal activities. This tends to be more prevalent among people who were active in Colombia, including refugees and migrants from higher social classes, people who feel part of the political system in the home country (whether as supporters or opponents of the government—see Bermudez, 2016). Flor, a young upper-middle class woman from Bogota who moved to Europe to further her postgraduate studies and lives in Madrid since 2003, talks about how her generation, having suffered different violence(s), is “very committed”. Being away from Colombia has made her more active defending her country and involved: “I believe it is like a debt that one has with the country… the minimum that those people away from the country can do is work harder for it”. In her narrative, Flor, a member of the Conservative party in Colombia (like her family) and supporter of the Uribe government (in power at the time), expresses the divisions and conflicts along class and ideology within the migrant community in Madrid, and how angry she is about the bad image of Colombia this presents. Partly in response to this, different Colombian governments have implemented policies aimed at engaging the diaspora politically ‘from above’, which, up to a point, has fuelled greater participation (Bermudez, 2014). For some politically-active Colombians abroad also, being freer from the threat of violence makes it easier to participate and ‘de-naturalise’ associations between politics and violence. Here, the experience of living in another democracy, less conflictive or corrupt, can be a major influence. This is the case for Nora, a young upper-middle class woman from Barranquilla who went to Europe to study (and get away from a relationship), arriving in London in 2004, with previous experience of direct involvement in party politics (Liberal party). For her, the heterogeneity of the Colombian migrant community, contrary to what Flor thinks, is an opportunity:

the important thing is that there are spaces for tolerance… in Colombia there is such a plurality of ideas because we are so diverse… that sometimes… unity is very difficult, but … if people could tolerate each other and talk… take advantage of the space here, in England, for instance, to do that, in a tranquil space where it is possible to discuss, where there are no bad ones, and we abandon [the idea of] the bad and the good… that your thinking is as worthy as mine.

The influence of contexts of settlement on diaspora or migrant transnational politics, in the case of Colombia, has been approached as well in other studies, for instance, in Argentina, highlighting the importance of ‘gaining distance’ from the situation in Colombia, but also of contact with other experiences of political violence and state terrorism (Hernández, 2012). Work has looked at how diasporas and transnational communities can be important for ending (or perpetuating) violence and conflict at home (Cochrane et al., 2009). The recent peace accords in Colombia are a good example. During the research in the UK and Spain in 2005–2007, at the time of Uribe’s democratic security agenda, with its emphasis on defeating the guerrillas militarily, and following the failure of previous peace processes, interviewees in Europe in general were wary of being able to influence what was happening in the home country. Even among those involved in collective initiatives and supporters of a negotiated end to the conflict, talk of divisions and lack of interest from a majority of migrants was prevalent (Bermudez, 2011). Still, the start of the new peace process under later president Santos generated new hopes and organised efforts from abroad (Martínez Leguízamo, 2017). These efforts were, for instance, key in including victims of the armed conflict abroad in the peace negotiations and in legislative initiatives such as Law 1448 (Law of Victims). During the 2016 peace plebiscite in Colombia, a slight majority of those who voted in Colombia said NO to the accords with the FARC (50.2%), compared to a majority who voted YES from abroad (54.1%). Although participation rates were low, 37% and 14% of those registered respectively, in Europe support for YES was higher, ranging between 68.5% (UK) and 96.3% (Norway) (Bermudez, 2021). Following the approval of the accords, the work done by organisations with victims of the violence in the diaspora, including the Truth Commission (CEV in its Spanish acronym), to bring to light the experiences of Colombians who left the country fleeing the conflict, has also opened up new spaces for breaking silences, generating trust and cooperation between different types of victims of the plural violence(s) affecting Colombia and migrants in general, as well as strengthening bridges with the home country. These initiatives offer a significant potential to create a non-violent, more democratic society (at home and abroad), as the group *Mujer Diáspora* (Women Diaspora), created in 2015, argues:

Along the last few decades, millions of men and women left Colombia looking for a better future… At a time when Colombia faces the task of rebuilding its historical memory to overcome a violent past and create a future in peace, the Colombia living abroad –the diaspora- feels the need to participate in this collective responsibility. The migratory process has equipped the diaspora with experiences, capacities and knowledge with a great potential to contribute to the transition to a more inclusive and democratic Colombia. (Conciliation Resources, 2017: 3).

## Conclusions

The tentative concepts of violent democracies and violent pluralism, based on different types of pervasive violence(s) committed by plural armed actors in pursuit of or to contest power, can be applied to Colombia, a country immersed in violence for most of its recent history. Although the focus has been on the armed conflict between the state, guerrillas and paramilitaries, political violence interrelates with drugs-related and other criminal and social violence(s). Thus, the recent peace accords with the FARC, the largest and oldest guerrilla in the country, generated new hopes of a more peaceful future. Colombians abroad, many of them displaced directly or indirectly by multiple violence(s), play a significant role in such a context. The narratives of the three groups of migrants and refugees interviewed in Europe over the last 2 decades exposed here contain multiple references to the violence(s) experienced in the home country and security gained in host societies despite facing many difficulties. As in Colombia, violence and other differences have contributed to mistrust, fear and weakened solidarities within the diaspora, complicating citizenship and place-belongingness, but they have also spurred individual and collective action to bring peace. This means both continuities and ruptures in silences and political behaviour, with some migrants remaining sceptical and averse to participating in what they consider a corrupt, violent democracy at home, while others see their new settings in the host society and recent transformations in Colombia as opportunities to do things differently. For refugees and others escaping violence, trust is harder to rebuild but at the same time their political capital draws them towards collective action; those who have always felt part of the system are also encouraged to continue their political involvement transnationally. On the other hand, among the excluded, place-belongingness can remain strong while practices of citizenship are weak.

The recent peace process created the space for the Colombian diaspora to make visible their experiences and work towards creating new spaces of tolerance and action to contribute to a more peaceful, democratic society, both in Colombia and abroad. However, the challenges are many. In the post-accords period violence continues to influence politics, with data for the 2019 local elections in Colombia showing levels of violence as high as previously, which in turn reduces electoral participation (Albarracín et al., 2020). Armed groups, political and criminal, continue to operate, and the increase in assassinations and threats against social and community leaders has led to new outflows of asylum seekers (Bermudez, 2021). This makes organised efforts from abroad towards peace and a more inclusive democracy at home harder, while at the same time the new spaces of tolerance and cooperation generated as a result of the peace process can provide support for new arrivals and people struggling for the same objectives in Colombia. An example of this is the numerous marches and other activities organised by Colombians in different countries abroad to denounce state repression of civil society in the latest protests against the government in Colombia (El Tiempo, 2021). With ample representation of young people, the messages broadcast in these marches abroad—“they are killing us”, “Colombia continues to resist”, “Far away but not absent”—suggest a new, more active diaspora intent on breaking the links between violence and politics and supportive of a more inclusive democracy. With new elections due in 2022, it remains to be seen if transnational migrant politics will continue to be focused more in the informal sphere, while the external vote continues low.

## Data Availability

Data sharing is not applicable to this article as no datasets were generated or analysed during the current study.

## Abbreviations

FARC: *Fuerzas Armadas Revolucionarias de Colombia*—Revolutionary Armed Forces of Colombia
UK: United Kingdom
UNHCR: United Nations High Commissioner for Refugees

## Footnotes

1 Own translation from the original in Spanish.

2 The Economist (2020): https://www.economist.com/graphic-detail/2020/01/22/global-democracy-has-another-bad-year.

3 Freedom House (2021): https://freedomhouse.org/country/colombia/freedom-world/2021.

4 https://www.un.org/development/desa/pd/content/international-migrant-stock.

5 https://www.unhcr.org/refugee-statistics/download/?url=xY4Nmu.

6 (Information anonymized).

7 All names used are pseudonyms to preserve anonymity. Quotations have been translated from the original in Spanish.
